# Structure determination of molecules in an alignment laser field by femtosecond photoelectron diffraction using an X-ray free-electron laser

**DOI:** 10.1038/srep38654

**Published:** 2016-12-09

**Authors:** Shinichirou Minemoto, Takahiro Teramoto, Hiroshi Akagi, Takashi Fujikawa, Takuya Majima, Kyo Nakajima, Kaori Niki, Shigeki Owada, Hirofumi Sakai, Tadashi Togashi, Kensuke Tono, Shota Tsuru, Ken Wada, Makina Yabashi, Shintaro Yoshida, Akira Yagishita

**Affiliations:** 1 Graduate School of Science, The University of Tokyo, 7-3-1 Hongo, Bunkyo-ku, Tokyo 113-0033, Japan; 2 College of Science and Engineering, Ritsumeikan University, 1-1-1 Noji-higashi, Kusatsu, Shiga 525-8577, Japan; 3 Quantum Beam Science Research Directorate, National Institutes for Quantum and Radiological Science and Technology, 8-1-7 Umemidai, Kizugawa, Kyoto 619-0215, Japan; 4 Graduate School of Advanced Integration Science, Chiba University, 1-33 Yayoi-cho, Inage-ku, Chiba, Chiba 263-8522, Japan; 5 Graduate School of Engineering, Kyoto University, Gokasho, Uji, Kyoto 606-8501, Japan; 6 Japan Synchrotron Radiation Research Institute, 1-1-1 Kouto, Sayo-cho, Sayo-gun, Hyogo 679-5198, Japan; 7 Graduate School of Science, Chiba University, 1-33 Yayoi-cho, Inage-ku, Chiba, Chiba 263-8522, Japan; 8 RIKEN SPring-8 Center, 1-1-1 Kouto, Sayo-cho, Sayo-gun, Hyogo 679-5148, Japan; 9 Institute of Materials Structure Science, KEK, 1-1 Oho, Tsukuba, Ibaraki 305-0801, Japan

## Abstract

We have successfully determined the internuclear distance of I_2_ molecules in an alignment laser field by applying our molecular structure determination methodology to an I 2*p* X-ray photoelectron diffraction profile observed with femtosecond X-ray free electron laser pulses. Using this methodology, we have found that the internuclear distance of the sample I_2_ molecules in an alignment Nd:YAG laser field of 6 × 10^11^ W/cm^2^ is elongated by from 0.18 to 0.30 Å “in average” relatively to the equilibrium internuclear distance of 2.666 Å. Thus, the present experiment constitutes a critical step towards the goal of femtosecond imaging of chemical reactions and opens a new direction for the study of ultrafast chemical reaction in the gas phase.

X-ray free-electron lasers (XFELs) are promising for determining atomically resolved structures and for tracing structural dynamics of molecules and nanoparticles with femtosecond time resolution^1^. Over the last decade, ground-breaking experiments on ultrafast X-ray diffraction (UXD) using the recently developed femtosecond XFELs, at the Linac Coherent Light Source^2^ at SLAC and SPring-8 Ångström Compact free-electron LAser (SACLA)^3^ at SPring-8, have been reported^4^,^5^,^6^,^7^. As an alternative to UXD, ultrafast X-ray photoelectron diffraction (UXPD) using XFELs provides a promising tool for investigating femtosecond structural dynamics because the photoionization cross sections of molecules are four to six orders of magnitude higher than those for X-ray scattering. Therefore, the UXPD method extends the time-dependent structure investigations of the UXD method to new classes of samples that are not accessible by any other method, e.g., dilute samples in the gas phase such as aligned, oriented, or conformer-selected molecules. To utilize this capability, several proposals^8^,^9^,^10^ and test experiments^11^,^12^,^13^,^14^ on the UXPD methods have been published. However, results on the transient structure of molecules during chemical reaction, which will be obtainable with the UXPD methods, have not yet been reported.

In the UXPD method, the gas-phase molecules must be aligned or oriented in space before the interaction with the XFEL to avoid averaging over all possible orientations. In the pioneering works on the UXPD method, sample molecules (C_8_H_5_F^11^,^13^, C_6_H_4_Br_2_^12^,^13^, and I_2_^14^) were adiabatically aligned by the electric fields of Nd:YAG lasers. The reported diffraction profiles for such molecules can be regarded as a snapshot of a “molecular movie” visualizing the femtosecond structural dynamics in a pump-probe experiment. Here, a fundamental question arises: whether the structure of a molecule in an intense alignment-laser field is the same as that in its ground state or not. To answer this question, we have applied the UXPD method to a simple I_2_ molecule to determine its structure ‒ in other words, its internuclear distance ‒ in the alignment-laser field.

In this Article, we report on the profile of I 2*p* photoelectron diffraction from I_2_ molecules with a higher degree of alignment compared with our previous work^14^, which was obtained using XFEL pulses from SACLA. Owing to the better alignment, we have succeeded in determining the average internuclear distance for the I_2_ molecular ensemble in alignment Nd:YAG laser fields by applying our molecular structure determination methodology^9^ to the newly observed I 2*p* photoelectron diffraction profile. Thus, we have established that the internuclear distance of I_2_ in the laser field is slightly elongated relatively to the equilibrium internuclear distance.

## Results

### Experimental setup and procedure

**Figure 1:**
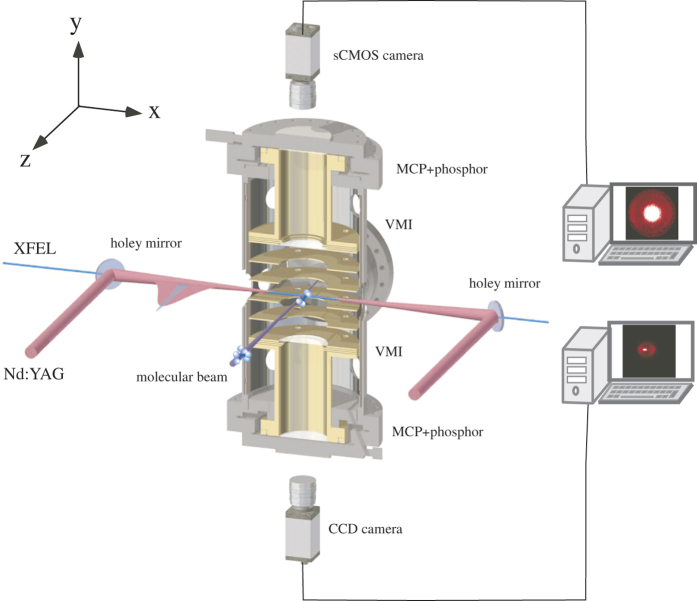
Schematic of the experimental setup. Two laser beams propagating along the x-axis in a collinear arrangement intersect a supersonic pulsed molecular beam along the z-axis at the centre of a vacuum chamber. A Nd:YAG laser is used to adiabatically align the sample I_2_ molecules that are probed by the XFEL. XPD images of the photoelectrons are recorded by the upper VMI. The degree of alignment is quantified using the 2D momentum distributions of the ionic fragments, which are registered by the lower VMI.

A pulsed supersonic molecular beam of sample I_2_ was introduced into the interaction region between facing velocity-map imaging spectrometers (VMIs) and was intersected by collinear pulsed lasers (Nd:YAG laser and XFEL)^14^ (see Fig. 1). The 10-ns-long pulses from the Nd:YAG laser adiabatically aligned the I_2_ molecules. The polarization vectors of the Nd:YAG laser and the XFEL were parallel to each other along the *z*-direction shown in Fig. 1. Here, alignment refers to the confinement of a molecular axis along the Nd:YAG laser polarization vector. Electrons produced by the XFEL pulses were accelerated towards the one VMI, which was operating in a velocity focusing mode, and then detected by a microchannel plate (MCP) detector backed by a phosphor screen. The two-dimensional (2D) electron images formed on the screen were recorded with a sCMOS camera and the data acquired for every single XFEL shot were read out by a personal computer (PC). Simultaneously, 2D ion images were measured with the other VMI and a detector system similar to that used for the electrons. From the 2D ion images, the degree of alignment of the I_2_ molecules was evaluated. The experiment was performed at the beamline BL3 in the experimental hatch EH4c of SACLA^3^,^15^. To analyse the XPD profiles within the theoretical frame work of a photoelectron diffraction model^9^, we selected the photon energy of the XFEL to be 4.7 keV, which is ~140 eV above the ionization threshold of I 2*p*_3/2_ (4.557 keV, ref. 16); thus, the kinetic energy *ε*_*p*_ of the I 2*p*_3/2_ photoelectrons was ~140 eV. The details of the experimental procedures are described in the Methods section.

### Electron and ion images from laser - aligned I_2_ molecules

**Figure 2:**
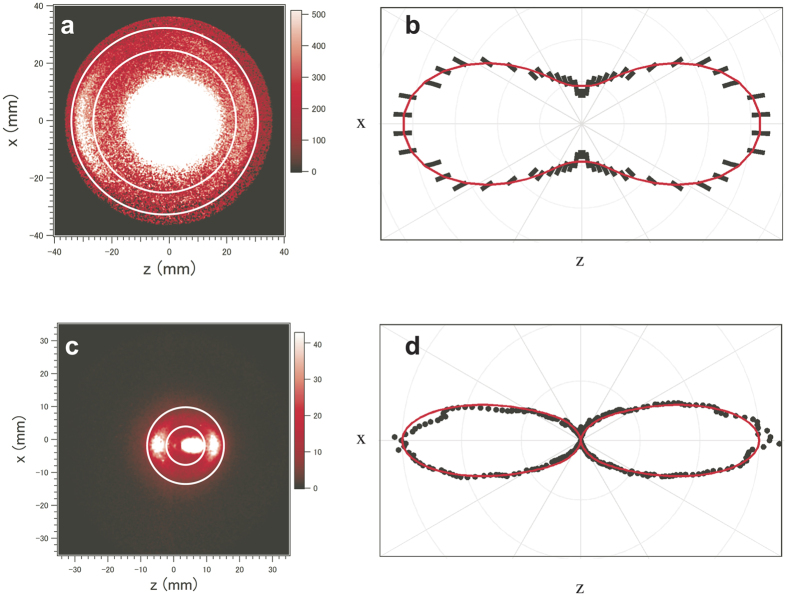
2D momentum images of electrons and ions and their polar plots. (**a**) The I 2*p* photoelectron image. The white circles indicate the radii of 26 and 30.5 mm to distinguish the central-ring image of low-energy electrons. (**b**) The I 2*p* XPD profile expressed as the polar plot. The short bars denote the statistical errors of the experimental data and the solid curve is the fitted result of the Legendre polynomials of F(*θ*_*e*_) ∝ *P*_0_(*θ*_*e*_) + 1.49*P*_2_(*θ*_*e*_) + 0.31*P*_4_(*θ*_*e*_) + 0.24*P*_6_(*θ*_*e*_). (**c**) Fragment-ion image indicating that the molecular axis distributions are aligned along the polarization vector of the Nd:YAG laser parallel to the z-axis. The white circles correspond to radii of 5 and 10 mm. (**d**) The molecular axis distributions expressed as the polar plot. The dots represent the experimental data, in which the background has been eliminated from the raw image and the solid curve P(*θ*) = cos^2^ *θ* + 1.82 cos^12^ *θ* is the result of the numerical simulation.

The 2D electron and ion images produced from the aligned I_2_ molecules using the Nd:YAG laser pulses are shown in Fig. 2(a) and (c), respectively. Each image was obtained by alternative measurements with and without the molecular beam and subtraction of the latter from the former. In the ion image, an intense central spot and an outer ring appear (see Fig. 2(c)). The central peak is created by both the atomic ions of He^+^ in the buffer gas and the molecular ions of I_2_^+^. The outer ring originates from the Coulomb-exploding fragment ions I^*n*+^ and is distributed along the polarization direction of the Nd:YAG laser pulses, parallel to the z-axis in the Fig. 2(c). The anisotropic distribution of the fragment ions is due to the alignment of neutral I_2_ molecules. Based on a numerical simulation for the 2D ion image, the most probable rotational temperature was estimated to be 5 K and the effective peak intensity of the Nd:YAG laser pulses was 6 × 10^11^ W/cm^2^ in the interaction region. These conditions resulted in a degree of alignment characterised by the alignment parameter^17^ <cos^2^ *θ* > = 0.734 ± 0.003, where *θ* is the angle between the molecular axis and the polarization direction of the Nd:YAG laser. Figure 2(d) shows the polar plot of the angular distribution of fragment ions with radii 5‒10 mm, which correspond to the charge states *n* of 4 ≤ *n* ≤ 6 (ref. 14). The details of the numerical simulation are provided in the Methods section.

The 2D electron momentum image in Fig. 2(a) consists of a central part, which originates from low-energy electrons via shake-off processes induced by Auger cascades, and the outer ring, corresponding to I 2*p* photoelectrons. The high kinetic energy (~140 eV) of the I 2*p* photoelectrons allows the distinction between the outer photoelectron ring and the intense low-energy central part. Figure 2(b) shows the polar plot of the angular distribution of the I 2*p* photoelectrons (detailed in the Methods section), which is hereafter referred to as the XPD profile. The XPD profiles of reflection-symmetric molecules, like the I_2_ molecule, that are aligned parallel to the polarization vector of the XFEL pulse, can be expressed by a series of even-order Legendre polynomials P_n_(*θ*_*e*_) (refs 18, 19, 20), where *θ*_*e*_ is the photoelectron ejection direction with respect to the molecular axis. In fact, the measured XPD profile is well reproduced by the Legendre polynomials of up to the 6th orders (see Fig. 2(b)). The contributions of higher order Legendre polynomials, which are responsible for the fine structure expected in the XPD profile, are smeared out owing to the imperfect alignment of the sample molecules. However, thanks to the higher degree of alignment of <cos^2^ *θ* > = 0.734 ± 0.003 compared to that of 0.61 ± 0.03 achieved in ref. 14, the intensity minima in the XPD profile are observed in the perpendicular directions to the polarization vector of the Nd:YAG laser; however, the fine structure due to photoelectron diffraction, as will be shown later, cannot be resolved. The improved XPD profile motivated us to analyse our new results on the basis of multiple-scattering XPD (MS-XPD) theory^9^ to extract the molecular structure of I_2_ in the 10-ns-long adiabatic- alignment Nd:YAG laser field.

### Molecular structure determination

We employed the muffin-tin approximation for molecular potentials, which considers spherical scattering potentials centred on each atom and a constant value in the interstitial region between atoms (as detailed in the Methods section). In this model, the photoelectron energy in the molecular region, *E*_*p*_, is described by *E*_*p*_ = *ε*_*p*_ + *V*_*0*_, where *ε*_*p*_ is the photoelectron kinetic energy measured from the vacuum level, and *V*_*0*_ is the energy between the vacuum level and the muffin-tin constant. For a given muffin-tin potential ‒ in other words, a certain molecular geometry ‒ we can calculate an XPD profile within the frame work of our MS-XPD theory^9^ for a given polarization geometry, in which the photoelectron energy *E*_*p*_ and the internuclear distance *R*_I-I_ are free parameters. Central photon energies of XFEL pulses fluctuate shot-by-shot, but their standard deviation is much smaller than the bandwidth Δ*E* (0.5%) at the photon energy of *E* = 4.7 keV^15^. Thus, due to this bandwidth of Δ*E*~24 eV, the photoelectron peak with the mean energy of *ε*_*p*_~140 eV has a width of | ± Δ*E*/2*|* × 2~24 eV (full width at half maximum). For convenience sake, we define a parameter range, Δ*E*_*a*_, for the photon energy, *E* = *V*_0_ + *ε*_*a*_, as Δ*E*_*a*_ = *V*_0_ ± Δ*E*. Under this definition, the parameter range of Δ*E*_*a*_ covers the possible range for the muffin-tin zero energy of *V*_0_.

In general, the XPD profiles are controlled by both the kinematical parameters on the polarization geometries and the dynamical parameters of *E*_*p*_ and *R*_I-I_. The former is given, but the latter is retrieved by the following procedure. First, we calculated the profiles for all geometries by using the set of two dynamical parameters, and constructed their weighed sum, considering the axis distribution of the sample I_2_ molecules (detailed in the Methods section). Finally, the weighed sum of the XPD profiles was convoluted over the experimental acceptance angles for the I 2*p* photoelectrons. To retrieve the internuclear distance information from the XPD profile calculated with this procedure, we performed a “trial-and-error” iterative procedure comparing the experimental XPD profile, *I*_exp_(*θ*), with the theoretical ones, *I*_theor_(*θ*), given by the set of two parameters, *E*_*p*_ and *R*_I-I_. The quality of the fit between the experiment and theory was evaluated by the reliability factor, or *R*-factor^9^, defined as


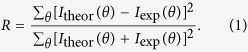


**Figure 3:**
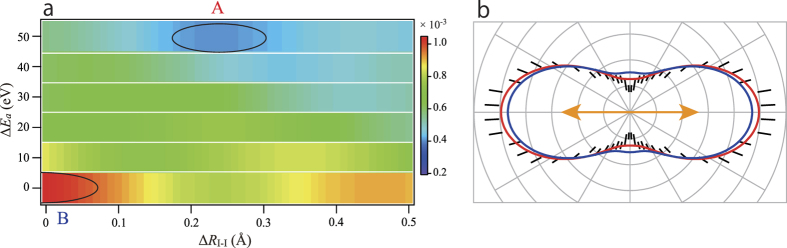
*R*-factor map as a function of parameters Δ*E*_*a*_ and Δ*R*_I-I_ (a) and relevant I 2*p* XPD profiles (b). In (**a**), a valley located in region A and a hill in region B. In (**b**), simulated XPD profiles at the minimum value of the *R*-factor in region A and at the maximum in region B are shown by red and blue curves, respectively. The experimental data are represented by the short bars, which are the same as those in Fig. 2(b).

The intensities *I*_theor_ and *I*_exp_ are normalized so that the area of each XPD profile is unity. Because *R* = 0 corresponds to the perfect agreement, we determined the minimum value of the *R*-factor to obtain the best solution for the internuclear distance. The *R*-factor map as a function of the two parameters is shown within an interesting parameter ranges of Δ*E*_*a*_ and Δ*R*_I-I_ in Fig. 3(a). The area (A) surrounded by the solid curve indicates the valley of the *R*-factor map. That is, the best solution for the deviation (Δ*R*_I-I_) from the equilibrium internuclear distance of 2.666 Å ranges from 0.18 to 0.30 Å, i.e., the internuclear distance is elongated by 0.18 to 0.30 Å. In parallel with this best solution the Δ*E*_*a*_ ranges from 45 to 55 eV. Referring to the value of *V*_0_ = 23 eV roughly evaluated by us (see the Method section), one gets Δ*E* ~ + 25 eV, which is comparable to the bandwidth of the photoelectron peak. Taking both the ambiguity of the *V*_0_ value and the photoelectron energy spread into account, the best solution of Δ*E*_*a*_~50 eV is rationalized. To illustrate the quality of the fit, the XPD profiles for the minimum and maximum values of the *R*-factor are depicted in Fig. 3(b) along with the experimental data. The best fitted curve reproduces the minima of the experimental XPD profile in the vertical direction. In contrast to this, the worst fitted curve makes the maxima in the direction. It is not surprising that one cannot see prominent differences between the best and worst fitted curves because the XPD profile averaged over the molecular axis distribution exhibits fairly simple structure, compared to the XPD profile for a given geometry (detailed in the Method section). The slightly insufficient fit between the XPD profile for the minimum value and the experimental data may be due to relativistic effects, which are discussed later.

Based on the above molecular structure determination methodology, we can conclude that the internuclear distance of the sample I_2_ molecules in the alignment Nd:YAG laser fields of 6 × 10^11^ W/cm^2^ is elongated by from 0.18 to 0.30 Å “in average” relatively to the equilibrium internuclear distance of 2.666 Å.

## Discussions

We have successfully determined the internuclear distance of I_2_ molecules in alignment laser fields by applying our molecular structure determination methodology, which is based on non-relativistic MS-XPD theory, to I 2*p* XPD profiles measured with femtosecond XFEL pulses. Consequently, we have revealed a bond softening of molecules in the alignment laser fields. This could be mainly because some portion of the I_2_ molecular ensemble is electronically excited via multi-photon processes of the Nd:YAG laser. Although the analysis of the experimental data relies on quantum computations, there is no doubt that the present experiment consists a critical step towards the goal of femtosecond imaging of chemical reactions and opens up a new direction in the study of ultrafast chemical reaction in the gas phase.

We further aim to improve the accuracy in the determination of the internuclear distance. The following two reasons are considered as the sources of the relatively large errors. i) The I 2*p* orbital is triply degenerated so that the I 2*p* XPD profiles obtained from the degenerated states are triply folded. More importantly, the I 2*p* XPD profiles are averaged over the axis distributions of the sample I_2_ molecules. As a result, the fine structures expected in the XPD profile are smoothed out owing to the axis distributions (see Fig. 5); however, we observed one maximum and one minimum in the XPD profile. ii) In the deep inner shells (like I 2*p*_3/2_ and I 2*p*_1/2_, which have binding energies 4557 eV and 4852 eV (ref. 16), respectively), the relativistic effects, which are not considered in our MS-XPD theory, are non-negligible. In fact, within a relativistic framework, the photoelectron asymmetry parameters for Sb 2*p*_3/2_ and 2*p*_1/2_ (which have binding energies 4137 eV and 4385 eV, respectively) were calculated as 1.23 and 0.97, correspondingly, at the photon energy 4509 eV (ref. 21). This implies that the difference of the asymmetry parameters is appreciable, although this difference must be reduced if these parameters are compared at the same photoelectron energy. Therefore, the XPD profile determined with our non-relativistic MS-XPD theory may not be fully reliable. Nevertheless, we consider that this issue is not critical, because although relativistic effects affect the primary photoelectron angular distributions, they do not influence the scattering in the molecules.

The above unfavourable conditions can be easily eliminated by using an XFEL in the soft X-ray region and measuring photoelectrons from non-degenerated *s* subshells with binding energies below approximately 3 keV. It must be noted that in these conditions, the photoelectron angular distributions are well described with a non-relativistic treatment^22^.

On the one hand, the structure of molecules in an intense (>10^14^ W/cm^2^), femtosecond optical laser pulse is known to change dynamically within the pulse duration via, e.g., bond softening^23^, Coulomb explosion^24^, and charge-resonance-enhanced ionization^25^. On the other hand, the structure of molecules in a moderately intense alignment pulse of the order of 10^12^ W/cm^2^ has been assumed to remain almost identical to that of the ground state, besides bending motions along the shallowest potential directions^26^. In contrast to this assumption, our present result demonstrates that the change of the internuclear distance, i.e., the excitation of the stretching motion along the relatively deep potential directions, is likely to be induced by the moderately intense alignment pulse. The ultrafast imaging^27^,^28^ of molecular orbitals by observing the spectrum of high-order harmonics attracts a lot of interests of chemists and physicists. The spectrum depends critically on both the shape of the molecular orbitals and the positions of the nuclei. Once the structure of a molecule aligned by an alignment pulse is determined by the XPD measurement, the images of the molecular orbitals that are associated with the deformed molecules can be obtained by retrieving the high-order harmonic spectra^29^,^30^. By comparing the orbital images of molecules aligned in the laser fields with those of molecules aligned in field-free conditions by non-adiabatic alignment^31^ or by plasma-shutter techniques^32^, we can investigate the correlations and couplings between electrons in the ground and excited states using the moderately intense laser fields.

## Methods

### Experimental details

The focused XFEL and Nd:YAG pulses were combined by a holey mirror as shown in Fig. 1. The XFEL pulses, with energy ~500 μJ/pulse and duration ~10 fs, were focused to a spot diameter of ~1 μm by Kirkpatrick-Baez (KB) mirrors^33^ located 100 mm downstream from the interaction region. The Nd:YAG pulses (Spectra Physics, Lab-530), with energy 800 mJ/pulse and duration ~10 ns, were focused to ~80 μm by a spherical lens placed outside the vacuum chamber. The spatial overlap of the XFEL and the Nd:YAG pulses was first examined by monitoring the images on a Ce:YAG phosphor screen in the interaction region and then confirmed by monitoring the degree of alignment of the sample molecules. The temporal overlap of the pulses was monitored by a fast photodiode. The Nd:YAG pulses were synchronized with the XFEL pulses by a pulse generator (Stanford Research, DG535), which was triggered by a master signal that delivers the XFEL pulses with a repetition rate of 30 Hz and triggered both the pulsed solenoid valve and the cameras.

A pulsed supersonic molecular beam was formed by expanding a gas mixture of the sample I_2_ molecules and 40-bar helium through the pulsed valve developed by Even and Lavie^34^ into the vacuum chamber. The valve was heated to 60 °C to provide a partial pressure of ~100 Pa for I_2_. The molecular beam passed through a 3-mm-diameter skimmer and was introduced into the interaction region, where the Nd:YAG and XFEL laser pulses were overlapped. The source and the main chamber were differentially pumped by turbo-molecular pumps and their typical pressures during the experiments were 1 × 10^−4^ and 2 × 10^−6^ Pa, respectively. The pulse duration of the valve was changed from 20.5 to 22 μs by monitoring the pressure of the source chamber. The pulsed valve was operated at a repetition rate of 15 Hz. The images (signal + background) with and those (background) without the sample molecules were alternately measured and then the images without the noise from the residual gas and the scattered XFEL were obtained by subtracting the background from the (signal + background) images. We acquired momentum-image data for 600,000 XFEL pulses, which correspond to 5.5 hours.

### Evaluation of degree of molecular alignment

The momentum image of the fragment ions, like that shown in Fig. 2(c), reflects directly the degree of alignment of the sample molecules. The raw image, however, contains some backgrounds partly due to traces of impurities in the molecular beam and partly to the inevitable dark currents, whose count rate is enhanced in the bright image obtained with the molecular beam. To efficiently eliminate such backgrounds, we simulated the angular distribution of the molecular axis by numerically solving the Schrödinger equation for a linear rotor. The effective Hamiltonian *H* is expressed in terms of dimensionless interaction parameters as^35^,^36^


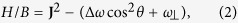



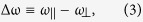


and


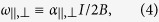


where **J**^2^ is the squared angular momentum operator, *I* is the intensity of the laser pulse, *B* is the rotational constant of the molecule, and *α*_*||*_ and *α*_⊥_ are the parallel and perpendicular to the molecular axis, respectively, polarizability components. The eigenfunctions of the Hamiltonian of Eq. (2) were averaged considering a Boltzmann distribution to obtain the spheroidal wavefunctions for the molecules with rotational temperature *T*_rot_. Here, we employed the intensity *I* of the laser pulse and the rotational temperature *T*_rot_ as the fitting parameters. Thus, we determined that both *I* = 6 × 10^11^ W/cm^2^ and *T*_rot_ = 5 K reproduced fairly well the experimental data. This numerical simulation of the molecular axis distribution resulted in the expectation value of cos^2^ *θ*,<cos^2^ *θ* > = 0.734 ± 0.003. Furthermore, we determined that the molecular axis distribution can be well represented by the simple functional form of P(*θ*) = cos^2^ *θ* + 1.82cos^12^ *θ*. Note that the intensity *I* can be regarded as the effective one, when the non-uniform laser intensity is averaged over the ionization volume of the XFEL pulses.

In the above evaluation of the degree of alignment for the sample molecules, we used the equilibrium internuclear distance of 2.666 Å in the ground state. To examine the internuclear distance effect on the degree of alignment, we assumed that all the molecules in the ensemble were elongated by 10% of the equilibrium internuclear distance. Then we estimated the degree of alignment for such molecular ensemble, in which the other conditions were set to the same as the beforementioned case. As a consequence of this, we obtained the degree of alignment of <cos^2^ *θ* > = 0.762 ± 0.003. The molecular axis distribution, which is used to construct theoretical XPD profiles, expected from this value for the degree of alignment was nearly the same as that for <cos^2^ *θ*> = 0.734 ± 0.003. The experimentally prepared molecular ensemble in the Nd:YAG laser are between the two extreme cases, i.e., all the molecules are either in the ground state or in the excited states. Therefore, the averaged degree of alignment over all the molecules in the ensemble must be between <cos^2^ *θ* > = 0.734 ± 0.003 and <cos^2^ *θ*> = 0.762 ± 0.003. From these considerations, we can conclude that the internuclear distance effect on the degree of alignment does not affect our molecular structure determination procedure with detectable amount.

### Data processing for the photoelectron polar plot

The positions of the detected electrons were determined offline by calculating the centre of the intensity weighted by the density of activated pixels, which provides a sub-pixel spatial resolution. The electron image shown in Fig. 2(a) has two components; the low-energy peak, which is associated with the Auger shake-off processes, and the high-energy ring of the I 2*p* photoelectrons. Although the I 2*p* photoelectron ring is almost separated from the intense low-energy peak, parts of the two peak components overlap with each other. To examine the XPD profile qualitatively, the radial distribution of the central part with radii 3‒20 mm was approximated with a Gaussian function by a least-squared procedure for every 6-degree sector from the polarization vector of the Nd:YAG laser. Then, the extrapolated tail component was subtracted from the electron signals in the region 26‒30.5 mm, where the I 2*p* photoelectron ring is located. Finally considering the symmetry restriction for the XPD profile, we averaged the I 2*p* photoelectron signals detected above and below the x-axis and those on the left and right of the z-axis to obtain the photoelectron polar plot.

### Muffin-tin potential

**Figure 4:**
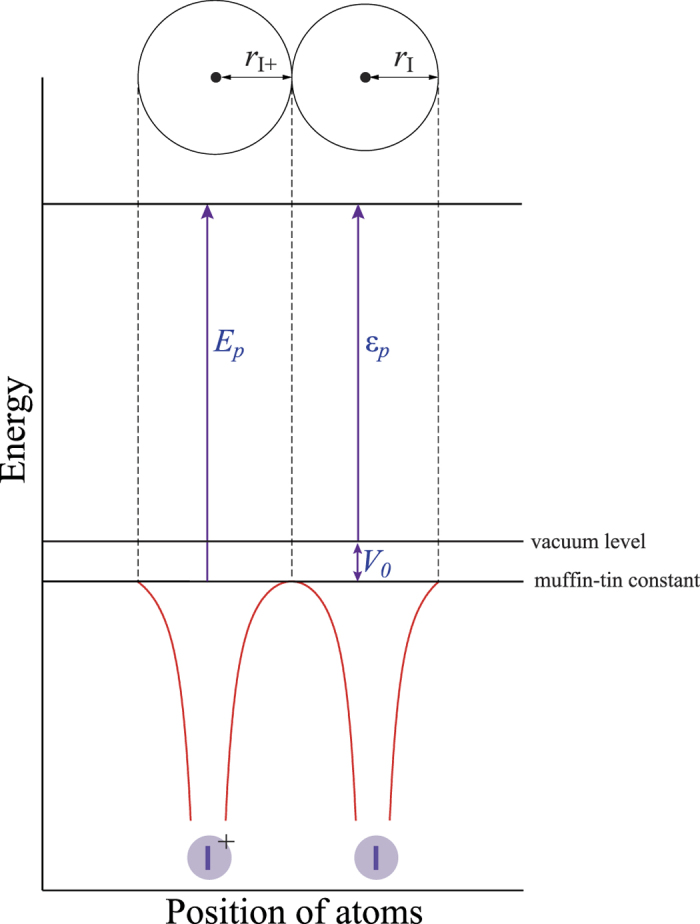
Schematic view of a one-dimensional muffin-tin potential of an I_2_ molecule. *ε*_*p*_: photoelectron kinetic energy from the vacuum level, *V*_*0*_: energy between the vacuum level and the muffin-tin constant, and *E*_*p*_: photoelectron energy in the molecular region. *r*_I+_ and *r*_I_ are the muffin-tin radius of the I^+^ ion and that of I atom, respectively.

A schematic of the one-dimensional muffin-tin potential considered for the I_2_ molecule is shown in Fig. 4. The muffin-tin constant is generally different from the vacuum level. Therefore, the photoelectron energy felt in the molecular region, *E*_*p*_, is described by *E*_*p*_ = *ε*_*p*_ + *V*_*0*_, where *ε*_*p*_ is the photoelectron kinetic energy measured from the vacuum level, and *V*_*0*_ is the energy between the vacuum level and the muffin-tin constant. We evaluated the muffin-tin radii and muffin-tin zero energy of *V*_*0*_ from each of the atomic potentials −*Z/r* + *V*_HF_, where the centre-of-gravity energy of *V*_HF_ was calculated using a Hatree-Fock program of Cowan^37^. Namely, we prepared two atomic potentials centred on the emitter I^+^ atom and the neighbouring I atom to determine the muffin-tin radii and *V*_*0*_. For the photoelectron emitter I^+^, an atomic potential with a core hole was calculated. The muffin-tin radii and *V*_*0*_ = 23 eV were determined from the intersection point of these two potentials, as the muffin-tin spheres do not overlap with each other. Although this muffin-tin model potential is very simple, it must be emphasized that we have confirmed that for *ε*_*p*_ > 100 eV, the XPD profiles calculated with this approach reproduce our relevant experimental data adequately^38^,^39^, and are in good accord with those obtained by more sophisticated density functional theory calculations^40^.

### XPD profile dependence on polarization geometries

**Figure 5:**
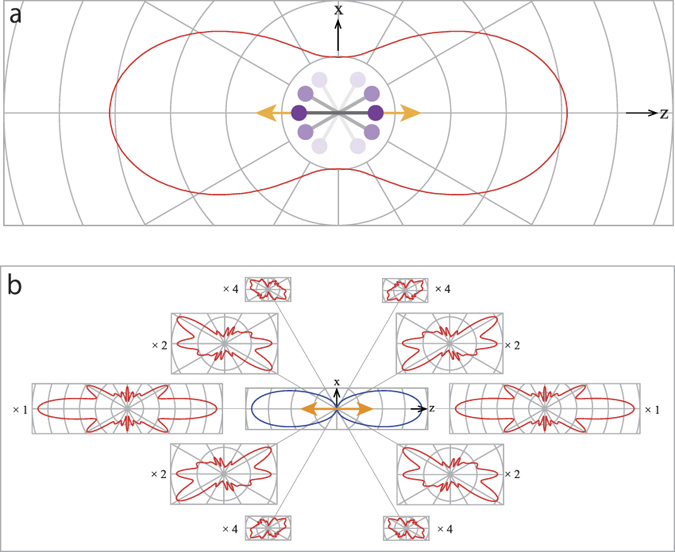
XPD profile integrated over the molecular axis distributions (a) and its decompositions (b). In (**a**), the reference axis of the XPD profile is the polarization vector of the XFEL, which is indicated by the double headed arrow. In (**b**), the central polar plot expresses the molecular axis distribution P(*θ*) = cos^2^ *θ* + 1.82 cos^12^ *θ*. The decomposed XPD profiles exhibit dramatic change depending on the geometries of the polarization vector of the X-rays and the molecular axis. Numbers on the left and right sides of the figures stand for their linear magnifications.

XPD profiles are affected by the geometry of the polarization vector of the X-rays and the molecular axis^18^,^19^,^20^. Therefore, the observed XPD profile of the laser-aligned molecules is the weighted sum of the XPD profiles over the molecular axis distributions that are described by the degree of alignment. This concept is depicted in Fig. 5(a) and (b). Figure 5(a) shows the measured XPD profile relatively to the polarization vector of the XFEL. Each component of the observed XPD profile, which is illustrated in Fig. 5(b), depends on the molecular axis because the polarization vector is fixed in the present experimental geometry. As expected, the molecular axis distributions strongly affect the profiles of the measured XPD profiles. In the extreme case of a fully random alignment, the XPD profiles cannot be measured but the photoelectron angular distributions can be observed relatively to the polarization vector of the X-rays.

Because both the polarization vector of the X-rays and the molecular axis are located on the xz plane in Fig. 5, the photoionization of the *p*_*x*_ and *p*_*z*_ orbitals contributes to the XPD profiles^14^. This geometry was selected for a convenient illustration and there are no restrictions to the photoionization of the *p*_*y*_ orbital. In fact, in our simulations, the three orbitals *p*_*x*_. *p*_*y*_and *p*_*z*_ were equally considered.

## Additional Information

**How to cite this article**: Minemoto, S. *et al*. Structure determination of molecules in an alignment laser field by femtosecond photoelectron diffraction using an X-ray free-electron laser. *Sci. Rep.* **6**, 38654; doi: 10.1038/srep38654 (2016).

**Publisher's note:** Springer Nature remains neutral with regard to jurisdictional claims in published maps and institutional affiliations.
